# Infectious Disease Detective: A Case of a Mysterious Rash

**DOI:** 10.7759/cureus.80999

**Published:** 2025-03-22

**Authors:** Lena DeLorenzo, Danielle C Thor, Cindy Hou

**Affiliations:** 1 Internal Medicine, Jefferson Cherry Hill Hospital, Cherry Hill, USA; 2 Internal Medicine, Jefferson Stratford Hospital, Stratford, USA; 3 Infectious Diseases, Jefferson Cherry Hill Hospital, Cherry Hill, USA

**Keywords:** dermatitis, dermatology, drug reaction, eosinophilia, infectious disease, internal medicine, perivascular dermatitis, rash

## Abstract

Drug reactions are often serious and complex events that can greatly affect patient outcomes. Many of these drug reactions manifest dermatologically by altering the skin’s structure or function and are most typically eosinophilic mediated. Fortunately, few drug reactions result in severe consequences for patients, and even fewer are fatal. However, the spectrum of what constitutes a drug reaction is expansive with consistent potential for novel findings. In the case presented, an 81-year-old male patient was found to have whole-body perivascular dermatitis with eosinophils eventually determined to be secondary to a delayed drug reaction. Through this report, the importance of detailed investigation of drug reactions is amplified in the context of rashes of unknown origin and beyond.

## Introduction

Adverse reactions to medications, or drug reactions, represent one of the most common immunological misfirings to presumably inert substances. Most drug reactions are eosinophilic in nature but could theoretically result from several points in the immune activation cascade. Immediate drug reactions are mediated by IgE, activating mast cells and basophils [1]. The activation of mast cells and basophils leads to the release of preformed histamine and tryptase, causing symptoms within a short period of time from exposure. A delayed drug reaction can present with variable cutaneous manifestations, including urticaria, vasculitis, and eosinophilia [2,3].

Historically, certain classes of medications are more prone to inducing drug reactions than others. These include antimicrobials, anti-inflammatories, and anticonvulsants, among others [4]. Furthermore, specific risk factors for drug reactions include polypharmacy, female sex, renal insufficiency, serious illness, liver disease, and active HIV and/or herpes infections [5, 6]. Lastly, with regard to routes of medication administration, increased risks of drug reactions are associated more with intravenous medications than oral and with chronic medications over single or limited doses [4].

The symptomatology of a drug reaction most often consists of maculopapular exanthema, observed in approximately 2% to 3% of patients hospitalized [7]. Drug-induced hypersensitivity syndrome may present with fevers, visceral involvement, and lymphocytosis [8]. These reactions can occur one to eight weeks post exposure and can have varying degrees of severity. More severe manifestations of drug reactions that carry high mortality rates include drug reaction with eosinophilia and systemic symptoms (DRESS), Stevens-Johnson, and acute generalized exanthematic pustulosis [9]. In this report, we describe an elderly patient who presented with a diffuse rash, as well as diarrhea, conjunctivitis, and hyponatremia. After ruling out a variety of infectious etiologies, a multidisciplinary care team concluded that the patient likely experienced a delayed drug hypersensitivity reaction. Through this case, we hope to reinforce the importance of a broad differential when assessing rashes of unknown origin to include drug reactions.

## Case presentation

**Figure 1:**
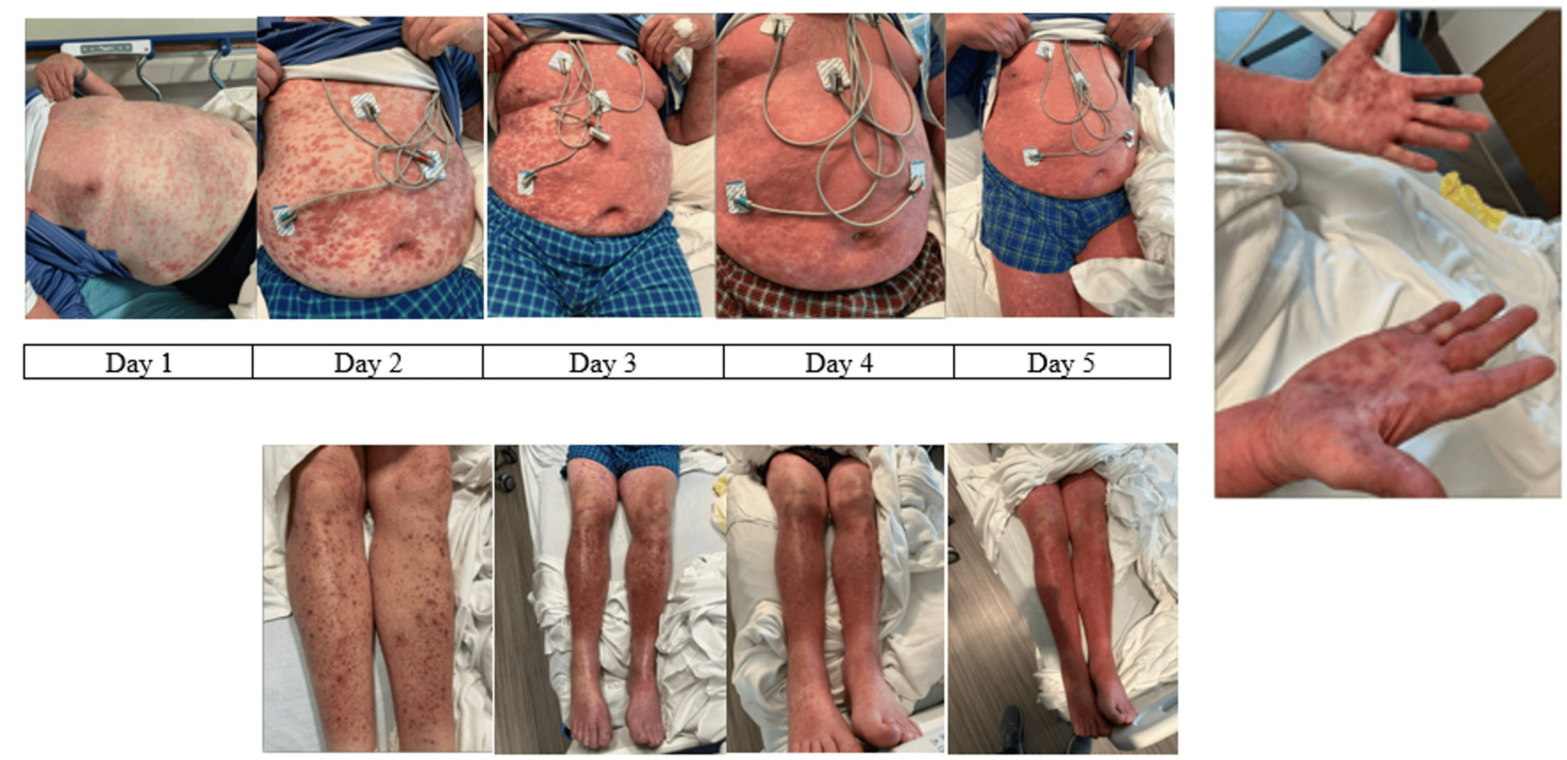
Pictures of the rash Note: Photographs of the lower extremity on day 1 are not available at the time of publication.

An 81-year-old male patient with a past medical history of atrial fibrillation (on anticoagulation), coronary artery disease, reduced ejection fraction heart failure, and insulin-dependent type 2 diabetes mellitus presented to his local emergency room with a chief complaint of a diffuse, pruritic rash. According to the patient, approximately two days prior to arrival, he was on a long-distance train ride from Florida to Massachusetts in July when he first felt a “lump” on the back of his neck. (Of note, the patient has a split residence between Florida and Massachusetts and utilizes long-distance trains to commute between the residences every year.) The patient originally dismissed the “lump” and did not seek further care until the presenting rash appeared. At the time of presentation, the lump resolved, and the rash quickly disseminated across his chest, back, and bilateral upper and lower extremities, including his palms, as well as his oral mucosa (Figure 1). In addition to the rash, the patient was experiencing rhinorrhea, low-grade fevers, generalized weakness, non-bloody diarrhea, and conjunctivitis. Of note, his wife, who was traveling with him, was asymptomatic throughout his clinical course. His rapid progression of symptoms prompted further evaluation in the emergency department.

Upon arrival, the patient was found to be hemodynamically stable with a temperature of 99.1°F but visibly uncomfortable due to the pruritic nature of the rash. Evaluation of his skin revealed a diffuse erythematous, maculopapular rash. His initial bloodwork, as listed in Table 1, demonstrated hypomagnesemia, an acute on chronic kidney injury, thrombocytopenia, elevated total bilirubin, and hyponatremia.

**Table 1:** The patient's bloodwork on admission GFR: glomerular filtration rate

Laboratory values	Result	Normal
Magnesium	1.0 mg/dL	1.3-2.1 mg/dL
Creatinine	1.88 mg/dL	0.50-1.20 mg/dL
Blood urea nitrogen	25 mg/dL	6-20 mg/dL
GFR	35	>=65
Sodium	129 mEq/L	133-145 mEq/L
Platelets	102	150-400
White blood cell count	11.4	3.7-10.9
Total bilirubin	2.2 mg/dL	0.0-1.0 mg/dL

**Figure 2:**
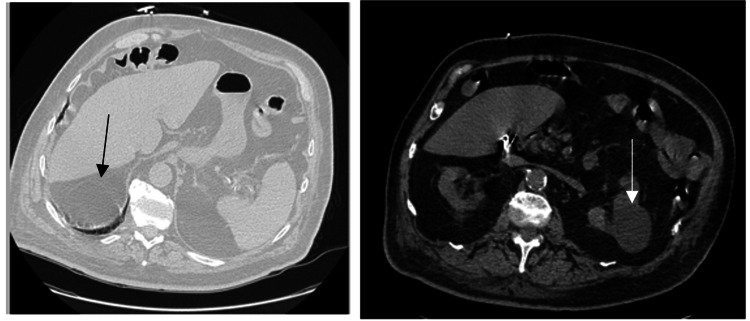
CT imaging of the abdomen and pelvis; the arrow highlights a liver cyst on the left and a renal cyst on the right

A computed tomography (CT) of the chest, abdomen, and pelvis obtained on admission (Figure 2) demonstrated left nephrolithiasis, a left renal cyst, a 2.4 cm liver cyst, suspicious of chronic pancreatitis, and colonic diverticulosis with no evidence of malignancy. The patient was ultimately admitted to the general medicine service for further workup and concerns for a possible active measles infection. 

On his admission, an extensive infectious diseases workup was obtained as detailed in Table 2. Given the rash’s palmar and wide involvement, the patient was placed on empiric doxycycline to cover for tick-borne illness. Additionally, the patient's frequent travel history to and from Florida made mosquito-borne illnesses a point of concern. However, this workup was ultimately consistently negative for any infectious source. Furthermore, the patient’s complete blood cell count differential was notable for an absolute eosinophil count of 1.40 B/L (0.00-0.70) and a percent eosinophils of 11% (0-6). Despite his empiric doxycycline treatment, his rash continued to progress. By the third day of his admission, his multidisciplinary care team began to seek alternative sources for his symptoms.

**Table 2:** Infectious diseases workup panel PCR: polymerase chain reaction

Infectious serology	Result
Dengue fever	Negative
West Nile virus	Negative
Zika virus	Negative
Chikungunya virus	Negative
*Babesia *PCR	Negative
*Ehrlichia *PCR	Negative
Treponema pallidum	Negative
Legionella	Negative
Rickettsia	Negative
Clostridium difficile	Negative
Stool PCR panel	Negative
Hepatitis panel	Negative
Coxsackie	Negative
Adenovirus	Negative
Herpes virus	Negative
Parvovirus	Negative
Rotavirus	Negative
Sapovirus	Negative
Norovirus	Negative
Respiratory syncytial virus	Negative
Rhinovirus	Negative
Rubeola	Negative

Ultimately, tickborne illnesses such as *Babesia*, *Ehrlichia*, and *Rickettsia *serologies were ruled out. Rapid plasma reagin (RPR) was ordered to rule out syphilis, and the results were negative. Given that the patient was hyponatremic, a *Legionella *urine test was ordered and the results were negative. Given the patient's persistent diarrhea prior to arrival, he was tested for *Clostridium difficile* and other infectious stool pathogens, all of which were negative. Various viral pathogens were ruled out, including hepatitis, coxsackie, adenovirus, herpes simplex virus (HSV), rotavirus, sapovirus, norovirus, influenza, respiratory syncytial virus (RSV), rhinovirus, and rubeola, all of which were negative. The parasitic workup was also negative. Initially given the patient’s presentation, measles was of concern; however, this was ruled out as well.

After an extensive review of the patient’s medical history and medication, a delayed drug hypersensitivity reaction to a chronic medication was suspected, as the patient endorsed no recent medication changes. The patient’s home medications included apixaban (Eliquis), clopidogrel (Plavix), dapagliflozin (Farxiga), sitagliptin (Januvia), rosuvastatin (Crestor), ezetimibe (Zetia), tamsulosin (Flomax), metoprolol, aspirin, and glipizide. Based on a literature review at the time of admission and the overall index of clinical suspicion, Januvia was perceived to be the offending agent. The hospital the patient was admitted to did not have an in-house dermatology team. However, an out-of-state dermatology affiliate was contacted and provided an unofficial recommendation to obtain a skin biopsy.

General surgery obtained a skin biopsy of the right thigh. The final pathology report read the results of this biopsy as "superficial perivascular dermatitis with eosinophils," which alluded to a differential diagnosis of a dermal hypersensitivity reaction to medication versus viral exanthema. Photographs of the biopsy were not available at the time of publication, due to a systematic error resulting in the corruption of the original photographs. Nephrology was also consulted due to concerns for nephrotic syndromes given his hyponatremia alongside his rash on admission. However, the lack of vascular involvement on this patient’s skin biopsy led them to also conclude his presentation was likely secondary to a drug reaction.

The patient’s primary medicine team, infectious disease team, and out-of-state dermatology team ultimately elected to re-approach this patient’s case as a delayed drug hypersensitivity reaction. He was treated with Solu-Medrol 1 g for three days, followed by a prednisone taper with marked improvement in his symptoms. He was eventually discharged with an extended steroid taper and levofloxacin for 14 days to complete his course. The patient followed up with infectious disease as an outpatient in Massachusetts. Infectious disease physicians following this patient had concerns for Steven Johnson's syndrome vs. toxic epidermal necrolysis given mucosal involvement, but our suspicions for either remained low. Following discharge, the patient had persistent right lower extremity cellulitis with bilateral lower extremity wounds that tested positive for *Enterobacter *in wound cultures. However, the patient has been lost to follow-up since.

## Discussion

In the case presented, an elderly male patient received an extensive infectious disease workup for what was inevitably attributed to a delayed drug hypersensitivity reaction. Although the initial concern for measles was not misguided given his presenting symptoms and travel history, his diffusely unremarkable workup demanded reevaluation. The similar concern for mosquito-borne illness was also valid given the patient’s travel history; however, his workup was equally unremarkable. 

Although uncommon, tolerance and/or intolerance to myriad medications can develop even after years of safe administration [10]. This patient’s medication list contained several examples of potential offending agents. Specifically, Farxiga and Januvia are both medications with “unspecified rashes” listed as potential side effects [11, 12]. Gupta et al. observed a female patient with a similar rash and presentation and biopsy demonstrating perivascular infiltrate with eosinophils following initiation of Januvia one week prior to her presentation [13]. The patient's Januvia and Farxiga were held at the time of intake due to institutional protocol. There are limited case reports of Januvia and Farxiga drug reactions; at the time of publication, all available case reports were reviewed. Based on relevant literature review at the time of diagnosis, it was determined Januvia would be the most likely offending agent. The patient was discharged after partial resolution of his rash; he was lost to follow-up to evaluate for complete resolution. The novelty of this case therefore lies in both the potential offending agent(s) and the chronicity of their administration versus other cases with shorter timelines.

The limitations of this case most notably include its single-patient focus and that the diagnosis of a drug reaction is typically a diagnosis of exclusion. Alternative pathology for this reaction may have been possible, particularly something viral in nature. Yet, the presentation above provides a relative degree of certainty of a drug-based source. Ultimately, this case is strengthened through its academic contribution to encourage clinician consideration of drug reactions higher in their differential of rashes of unknown origin.

## Conclusions

This case report highlights the necessity of consideration for delayed drug hypersensitivity reactions within the differential diagnosis of rashes of unknown origin. Through this attention, clinicians will hopefully grow to appreciate the breadth of this presentation and the medications that may cause it.
